# High-Efficiency Secretion and Directed Evolution of Chitinase BcChiA1 in *Bacillus subtilis* for the Conversion of Chitinaceous Wastes Into Chitooligosaccharides

**DOI:** 10.3389/fbioe.2020.00432

**Published:** 2020-05-07

**Authors:** Sijia Wang, Gang Fu, Jinlong Li, Xunfan Wei, Huan Fang, Dawei Huang, Jianping Lin, Dawei Zhang

**Affiliations:** ^1^Key Laboratory of Zoological Systematics and Evolution, Institute of Zoology, Chinese Academy of Sciences, Beijing, China; ^2^University of Chinese Academy of Sciences, Beijing, China; ^3^Tianjin Institute of Industrial Biotechnology, Chinese Academy of Sciences, Tianjin, China; ^4^Key Laboratory of Systems Microbial Biotechnology, Chinese Academy of Sciences, Tianjin, China; ^5^Biodesign Center, Tianjin Institute of Industrial Biotechnology, Chinese Academy of Sciences, Tianjin, China; ^6^College of Life Sciences, Nankai University, Tianjin, China; ^7^College of Pharmacy, Nankai University, Tianjin, China

**Keywords:** chitin, chitinase, BcChiA1, high-throughput screening, synergistic effect, BatLPMO10, chitooligosaccharide

## Abstract

Limitations of enzyme production and activity pose a challenge for efficient degradation of chitinaceous wastes. To solve this problem, we engineered a system for high-yielding extracellular secretion of chitinase A1 from *Bacillus circulans* (BcChiA1) in *B. subtilis*. Furthermore, an innovative chitinase high-throughput screening method based on colloidal chitin stained with Remazol Brilliant Blue R (CC-RBB) was established and used to identify three mutants with improved chitinase activity: Y10A/R301A/E327A (Mu1), Y10A/D81A/E327A (Mu2), and F38A/K88A/R301A (Mu3). Their highest specific activity reached 1004.83 ± 0.87 U/mg, representing a 16.89-fold increase in activity compared to native BcChiA1. Additionally, we found that there is a synergistic effect between BcChiA1 and a lytic polysaccharide monooxygenase from *Bacillus atrophaeus* (BatLPMO10), which increased the chitin processing efficiency by 50% after combining the two enzymes. The yield of chitooligosaccharide (COS) production using the mutant Mu1 and BatLPMO10 reached 2885.25 ± 2.22 mg/L. Taken together, the results indicated that the CC-RBB high-throughput screening method is a useful tool for chitinase screening, and evolution of BcChiA1 in collaboration with BatLPMO10 has tremendous application potential in the biological treatment of chitinaceous wastes for COS production.

## Introduction

Chitin is an insoluble, linear, high molecular weight polymer composed of β-1,4 linked *N*-acetylglucosamine (GlcNAc) units, and is one of the most ubiquitous polysaccharides in nature, second only to cellulose (Singh et al., 2017). It is the major structural component of a wide range of organisms, most notably fungi and arthropods, including species of shrimp and other seafood (Kumar et al., 2018). It has been reported that the worldwide seafood industry generates approximately 6–8 million tons of shell-waste annually (Gao et al., 2016). However, there is a limited scope of commercial applications of chitin despite its great quantity and notable biochemical characteristics, mainly due to its high crystallinity and insolubility which make processing a challenge (Kumar et al., 2018). Since the degradation of chitin is a time-consuming and arduous task, disposal of chitin-containing waste has become an environmental challenge and is on the verge of becoming a crisis for many countries. Therefore, it is imperative to find an effective and environmentally friendly means to degrade chitinaceous wastes, and preferably valorize it for use in valuable products. Chitooligosaccharides (COS) are chitin derivatives with a low degree of polymerization (≤20) that have great value due to their improved solubility (Liang et al., 2018). Moreover, they are highly biocompatible and non-toxic, and possess reported antiviral, antibacterial, antifungal, immunoregulatory, and antioxidant activities (Liaqat and Eltem, 2018). Hence, COSs have multiple promising applications in various fields such as food processing, biomedicine, agriculture, water purification, and cosmetics (Naveed et al., 2019).

Enzymatic hydrolysis of chitin is a good substitute for chemical treatments. As specific enzymes that convert chitin into COS, chitinases (EC 3.2.1.14) play a pivotal role in chitin biodegradation by catalyzing the hydrolysis of the β-1,4-glycosidic bonds that form chitin. Several chitinases have been isolated and characterized from various sources. *Bacillus circulans* WL-12 was found to secrete at least six chitinases, among which chitinase A1 (BcChiA1) exhibited the highest affinity and hydrolytic activity toward crystalline chitin (Watanabe et al., 2001; Ferrandon, 2003). More importantly, the catalytic mechanism of BcChiA1 has been thoroughly studied (Nakamura et al., 2018), which lays a solid theoretical foundation for follow-up research that can explore the evolution and optimization of BcChiA1.

However, the practical application of these enzymes remains challenging due to the low levels of production and weak activity of chitinases in wild type strains, which limits efforts to break down crystalline chitin. Heterologous expression is an effective strategy to increase the production of target proteins. As one of the most widely used expression systems, *B. subtilis* possesses many advantages such as a high growth rate, short fermentation period, easy genetic manipulation, and superior secretion capacity of recombinant proteins (Cui et al., 2018; Gu et al., 2018; Cai et al., 2019). Moreover, as one of the most widespread and powerful strategies for protein design, directed evolution plays a key role in enhancing the activity of target enzymes through the construction of mutant libraries and high-throughput screening (Porter et al., 2016). For example, the catalytic efficiency of a chitinase from *Beauveria bassiana* was enhanced 2.7-fold (Fan et al., 2007), while that of an improved chitinase from *Bacillus licheniformis* was 2.7-fold higher than that of the wild type (Songsiriritthigul et al., 2009). Despite these promising early efforts, the current methods of directed evolution have not yielded optimal results in chitinases, and there is still much room for innovation and improvement. Therefore, we created a new screening method based on using colloidal chitin stained with Remazol Brilliant Blue R (CC-RBB) as a substrate to probe chitinase activity. Furthermore, we found explanations for the evolved mutants of BcChiA1 by computing high-energy intermediate (HEI) states, in that the states observed in computational simulations can reflect the catalytic activity of enzyme variants (Bolon and Mayo, 2001; Hermann et al., 2006; Nanda and Koder, 2010; Grisewood et al., 2013; London et al., 2015). Bell and Koshland (1971) demonstrated that covalent enzyme-substrate intermediates participate in enzymatic reactions. The covalent intermediate state is a high-energy or transition state between the ground state and the product state. Lahiri et al. (2003) found that the stabilization of HEI states along the reaction trajectory is the crucial determinant behind the catalytic ability of enzymes.

Improving substrate pretreatment efficiency is also a vital prerequisite to biocatalytic processes. Recent studies have revealed that lytic polysaccharide monooxygenases (LPMOs) can oxidize polysaccharides with high degrees of crystallinity at diverse carbon positions to increase substrate accessibility to glycoside hydrolases (Vaaje-Kolstad et al., 2010; Isaksen et al., 2014). Some studies have shown that the pretreatment of chitin with LPMOs led to increases in product yields between 2.49- and 3-fold compared with chitinase treatment alone (Manjeet et al., 2013; Yang et al., 2017). Here, BatLPMO10, which was identified from a screen of fermented food, was used for chitin pretreatment to maximize the contact between the chitin substrate and BcChiA1, and to further enhance the production of COS.

In this study, we sought to overcome the limitations of catalytic activity and production of wild-type BcChiA1 by establishing a heterologous expression system in *B. subtilis* and performing directed evolution of this enzyme. In addition to optimizing the properties of BcChiA1 itself, we wanted to understand whether there is a synergistic effect between BcChiA1 and BatLPMO10 in the biodegradation of chitin. Thus, our objective is not only to achieve the highly efficient degradation of chitinaceous wastes, but also to obtain valuable COS products with high yield.

## Materials and Methods

### Bacterial Strains, Plasmids and Culture Conditions

The bacterial strains and plasmids used in this study are listed in the Supplementary Material. *E. coli* DH5α was used for molecular cloning and plasmid construction, while *B. subtilis* 1A751 served as the expression host for BcChiA1 and BatLPMO10. All the bacterial strains were cultured in Luria-Bertani medium at 37°C and 220 rpm. The plasmid pMATE (Yue et al., 2017) was used as the expression vector for BcChiA1 and BatLPMO10.

### Plasmid Construction

The primers and the signal peptides used in this study are listed in the Supplementary Material. The genes encoding BcChiA1 from *Bacillus circulans* (GenBank: AAA81528.1) and BatLPMO10 from *Bacillus atrophaeus* (GenBank: ADP32663.1), both without the native signal peptide and with a C-terminal his-tag, were codon optimized and synthesized by GENEWIZ (Suzhou, China). The plasmid backbone of pMATE including the promoter P*_*malA*_* is from our laboratory. SP*_*phoD*_*, SP*_*lipA*_*, SP*_*ywbN*_*, SP*_*nprE*_*, SP*_*sacB*_*, and SP*_*yvcE*_* (SP: signal peptide) were obtained by PCR using the genome of *B. subtilis* 168 as the template. All the DNA fragments were amplified using PrimeSTAR polymerase (Takara, Dalian, China). Construction of the recombinant plasmids was entirely in accordance with the operating instructions of the ClonExpress^®^ II One Step Cloning Kit (Vazyme Biotech Co., Ltd, Nanjing, China). The basic molecular cloning techniques were described in the literature (Spizizen, 1958; Sambrook et al., 1989; Smith, 1991). Based on the resistance markers on the plasmids, the transformants were selected on Luria-Bertani agar plates containing 100 μg/mL ampicillin for *E. coli* or 20 μg/mL kanamycin for *B. subtilis.*

### Shake-Flask Fermentation Experiments and SDS-PAGE Analysis

The recombinant strains were streaked onto Luria-Bertani agar plates containing 20 μg/mL kanamycin and cultivated overnight at 37°C. Then, a single colony was used to inoculate fresh Luria-Bertani medium as the seed for further fermentation. The resulting seed cultures were transferred at 1% inoculum size into 30 mL of 2 × SR medium (30 g/L tryptone, 50 g/L yeast extract and 6 g/L K_2_HPO_4_, pH 7.2) with 50 μg/mL kanamycin in 250 mL shake flasks, and cultivated at 37°C and 220 rpm for 48 h. After inoculation, 30 g/L final concentration of maltose was added from a 500 g/L stock solution as inducer immediately.

After 48 h of fermentation, the supernatant components of the samples were collected by centrifugation (4°C, 14,000 *g*, 10 min). The cell precipitate was resuspended in 50 μl lysis buffer (50 mM Tris-HCl, 2.5 mM EDTA, pH 8.0) per one unit of OD_600_. After adding 1:5 volume of 5 × SDS-PAGE sample loading buffer [250 mM Tris-HCl, 10% (v/v) SDS, 0.5% (w/v) bromophenol blue, 50% (v/v) glycerol, and 5% (w/v) β-mercaptoethanol, pH 6.8], the samples were boiled for 20 min. SDS-PAGE was carried out on NuPAGE 10% Bis-Tris protein gels (Invitrogen Life Technologies, CA, United States) to investigate the expression of BcChiA1 and BatLPMO10. The gels were stained with Coomassie Brilliant Blue R-250 (Solarbio life sciences, Beijing, China), and ColorMixed Prestained Protein Marker (Solarbio Life Sciences, Beijing, China) was used to evaluate the approximate molecular weight of the target proteins.

### Enzyme Purification and Chitinase Activity Assay

The purification of BcChiA1 and BatLPMO10 was conducted at 4°C. After shake-flask fermentation, the crude enzymes were collected by centrifugation at 18,693 × *g* for 20 min and subsequently loaded onto the affinity columns (1.5 × 8 cm) containing 5 ml Ni NTA Beads 6FF (Solarbio Life Sciences, Beijing, China) and allowed to bind for 2 h. The beads were washed with buffer A (50 mM sodium phosphate, pH 7.6), followed by a gradient of buffer B (50 mM Tris-HCl, 500 mM NaCl, 50–300 mM imidazole) to wash out the impurities and weakly bound proteins. BcChiA1 and BatLPMO10 were eluted with buffer C (50 mM Tris-HCl, 500 mM imidazole and 500 mM NaCl) and the volume was reduced to 2 ml by ultrafiltration through 10 kDa and 3 kDa molecular weight cutoff membranes (Millipore, United States), respectively. The two purified enzymes were verified by SDS-PAGE and their concentrations were determined using the BCA Protein Quantitation Kit (Solarbio Life Sciences, Beijing, China).

A sensitive and simple method based on the substrate CC-RBB was used to evaluate the chitinase activity as described previously (Gómez Ramıìrez et al., 2004). A mixture comprising 1 ml of the enzyme solution and 1 ml of 40% (w/v) CC-RBB suspension was incubated at 50°C for 1 h. Enzymes deactivated by boiling in a water bath for 10 min were used as negative controls. All the tubes were centrifuged at 12396 × *g* for 5 min, and the absorption of the supernatants was measured at 595 nm. One unit (U) of chitinase activity was defined as the amount of enzyme that causes an increase of 0.01 in the absorbance under the above conditions. The protein concentrations of the enzyme samples were determined as described above.

### Microstructure Analysis of Chitin Treated With BcChiA1

The morphology of the treated chitin was analyzed by scanning electron microscopy (SEM) using a HT7700 Exalens (Hitachi, Tokyo, Japan). Samples were dehydrated using the critical point drying method and fastened to a metal stub using conductive carbon tape. The chitin was sprayed with a platinum film (Pt coated) and observed at an acceleration voltage of 15 kV (Huang et al., 2018).

Fourier Transform Infrared Spectroscopy (FT-IR) was carried out on a Nicolet IS5 670 (Nicolet Tech, Wisconsin, United States) within a range of 4000 cm^–1^ to 400 cm^–1^ based on the attenuated total reflection method (Zhang et al., 2018).

The X-ray diffraction (XRD) measurements were conducted on a Bruker D8 Advance diffractometer (Bruker, Karlsruhe, Germany) with Cu Kα radiation (λ = 1.5418 Å). The data on the X-ray powder diffraction of chitin was obtained in a range of 2θ from 5° to 60° in steps of 0.02°. The crystallinity index (I_*CR*_) of the chitin samples was calculated according to the formula I_*CR*_ (%) = (I_110_-I_*am*_)/I_110_ × 100%, where I_110_ refers to the peaks at 2θ ≈ 20°, corresponding to the maximum intensity, and I_*am*_ represents the intensity of the amorphous diffraction at 2θ≈16°, as described before (Zhang et al., 2018).

### Synergistic Effects of BcChiA1 and BatLPMO10 on the Degradation of Chitin

To determine the synergistic action of BcChiA1 and BatLPMO10, samples comprising 500 μl of purified BatLPMO10 (9, 18, 36, 72, 144, or 288 nmol), 1 ml of 40% CC-RBB in sodium phosphate buffer (50 mM, pH 7.0) and 1 mM ascorbic acid were incubated at 50°C for 1 h. Enzymes deactivated by boiling in a water bath for 10 min were used as negative controls. Then, 500 μl of purified BcChiA1 (9 nmol) was added and the mixtures were incubated for another 1 h at 50°C. All the enzyme reactions were conducted under constant shaking at 200 rpm. After that, the reaction mixture was centrifuged at 12396 × *g* for 5 min, and the absorbance of the supernatants was measured at 595 nm. All reactions were performed in triplicate.

### High-Performance Liquid Chromatography

The degradation products of chitin were analyzed via HPLC on a Waters 600 instrument (Waters Corp., Milford, MA, United States) equipped with an NH_2_ Column (5 μm, 4.6 mm × 250 mm; Shimadzu, Kyoto, Japan) and an ultraviolet detector set at 195 nm. A reaction system containing 1U purified chitinase and 1 ml 20% CC-RBB solution was incubated at 50°C and 200 rpm for 24 h. The samples were filtered through a 0.22 μm pore-size membrane. The mobile phase was composed of acetonitrile and water (70:30, v/v) at a flow rate of 0.5 ml/min, and the injection volumes was 10 μl. The products were quantified by comparing to standard curves made using standard COS samples with degrees of polymerization 1-6 (BZ Oligo Biotech Co. Ltd, Qingdao, China).

### Directed Evolution of BcChiA1

Error-prone PCR was applied to introduce random mutations into the BcChiA1 gene (1.9 kb) using the Adjustable Error-prone PCR kit (Tiandz, Beijing, China). PrimeSTAR polymerase was used to clone the vector backbone of the pMATE plasmid. The DNA multimer was produced by prolonged overlap extension PCR (POE-PCR) with PrimeSTAR polymerase based on two DNA templates containing 3’ and 5’ overlapping termini of 40-50 bp. Finally, the multimers were used to transform competent *B. subtilis* 1A751. The transformants were screened on Luria-Bertani agar plates containing 20 μg/mL kanamycin. Individual bacterial colonies from the mutant library were seeded into 96 deep-well plates containing 0.5 mL Luria-Bertani medium with 20 μg/mL kanamycin, 2% CC-RBB (w/v) and 1% maltose per well. The plates were cultivated at 37°C and 800 rpm for 24 h on a Microtron 28759 shaker (Infors, Bottmingen, Switzerland), and then centrifuged on a JXN-26 centrifuge (Beckman, CA, United States) at 18923 × *g* for 30 min. The culture supernatants were transferred to corresponding wells of 96-well ELISA plates by an RV-3S-S11 Automatic Liquid Handling Workstation (Caliper Life Sciences, Boston, United States) and the absorbance at 595 nm was recorded for each well.

### Molecular Dynamic Simulations

**FIGURE 1:**
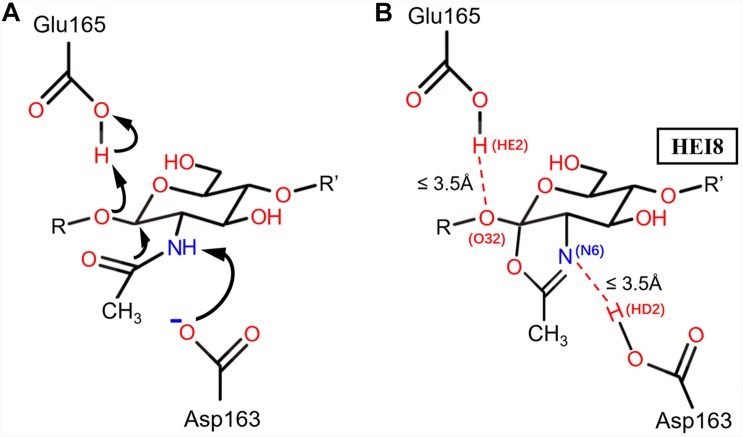
The catalytic mechanism of BcChiA1 and the HEI state of the substrate bound to BcChiA1. R and R’ represent the (GlcNAc)s that connect to the O atoms on C1 and C4, respectively. **(A)** Proposed mechanism of substrate-assisted catalysis by BcChiA1. **(B)** The HEI state of (GlcNAc)_8_, designated as HEI8. The red dotted line indicates the distance of proton transfer, which forms a new pentacyclic structure.

Since chitin is a high-molecular-weight polymer with poor solubility, which presents difficulties in docking simulations with chitinase, we chose octa-*N*-acetyl-chitooctaose,(GlcNAc)_8_, as the substrate to reflect the real-life conditions as closely as possible. (GlcNAc)_8_ was used to simulate the chitin hydrolysis reaction based on a published crystal structure (PDB ID: 1EIB) (Papanikolau et al., 2001). A HEI state substrate (Bolon and Mayo, 2001; Hermann et al., 2006; Hermann et al., 2007; Grisewood et al., 2013), designated as HEI8 (Figure 1), was generated according to the reaction mechanism (Nakamura et al., 2018). The initial wild-type (WT) complex was constructed using the glide module with ligand sampling refine only in Schrödinger software, with restriction between BcChiA1 and HEI8. The 3D complex models of Mu1, Mu2, and Mu3 were built based on the WT complex using ROSETTA3 enzyme design (Leaver-Fay et al., 2011; Richter et al., 2011), and the detailed parameters are shown in the Supplementary Material. AMBER16 was used for the energy minimization of the constructed model and molecular dynamics (MD) simulation of the final model using the ff14SB.redq force field. The constrained MD simulations with the HEI state of WT-HEI8, Mu1-HEI8, Mu2-HEI8 and Mu3-HEI8 as initial input were conducted for 10 ns (Case et al., 2016). Finally, 100 ns MD simulations were performed without any restriction. The complete simulation methodology used in this work is available in the supporting information. The MD trajectories were applied for further analysis and used to determine relevant binding poses.

## Results and Discussion

### Efficient Secretory Expression of BcChiA1 and BatLMPO10 in *B. subtilis*

**FIGURE 2:**
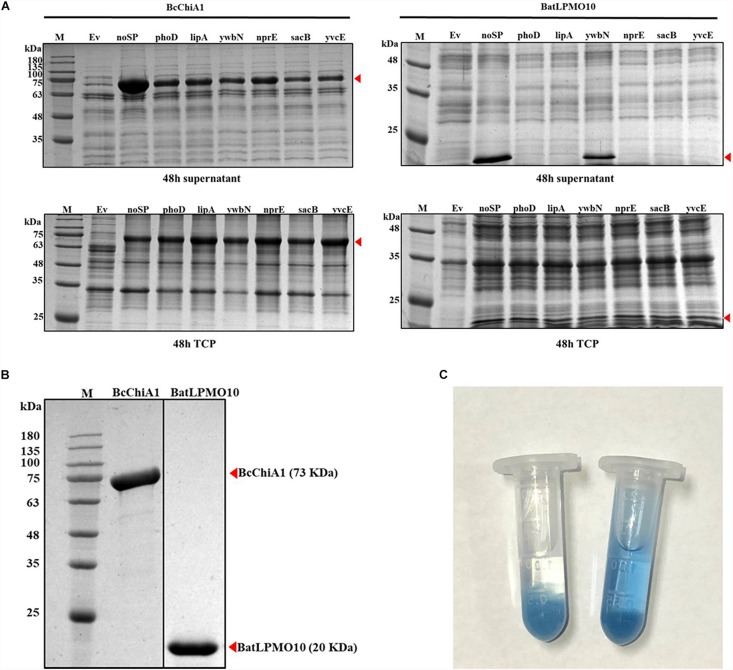
SDS-PAGE analysis and enzyme activity assay. **(A)** Comparison of the supernatant and total cell protein of cells expressing BcChiA1 and BatLPMO10 fused with different signal peptides. Column M, molecular weight marker; Ev, empty pMATE plasmid in strain 1A751. From left to right: noSP, SP*_*phoD*_*, SP*_*lipA*_*, SP*_*ywbN*_*, SP*_*nprE*_*, SP*_*sacB*_* and SP*_*yvcE*_*. **(B)** SDS-PAGE analysis of BcChiA1 and BatLPMO10 purified by his-tag affinity chromatography. **(C)** Determination of BcChiA1 activity with CC-RBB as the substrate (left) deactivated BcChiA1; (right) active BcChiA1.

In this study, *B. subtilis* 1A751 was used as the expression host and plasmid pMATE was used for the expression of BcChiA1 and BatLPMO10. We verified the effect of six different signal peptides (SP*_*phoD*_*, SP*_*lipA*_*, SP*_*ywbN*_*, SP*_*nprE*_*, SP*_*sacB*_*, and SP*_*yvcE*_*) from *B. subtilis*, as well as a construct without a signal peptide (noSP) on the secretion of the two enzymes. After 48 h of shake-flask fermentation, bands corresponding to the target proteins were clearly visible on SDS-PAGE gels (Figure 2A). The apparent molecular weights of BcChiA1 and BatLPMO10 in this study were approximately 73 and 20 kDa, which was in agreement with previous reports (Watanabe et al., 1990; Yu et al., 2016). The results indicated that all signal peptides led to successful BcChiA1 secretion, but the noSP group performed best. For BatLPMO10, the noSP group had the best performance, followed by SP_*ywbN*_, while little or no BatLPMO10 secretion was observed with the other tested signal peptides. In general, signal peptides are crucial for the transport and secretion of target proteins. Nevertheless, an inappropriate signal peptide can have a negative influence on the yield and secretion of target proteins (Nguyen et al., 2018). Fortunately, we achieved highly efficient secretion of both target proteins without extensive signal peptide screening. In addition, *B. subtilis* has an intrinsically superior secretion capacity, which makes the subsequent process of separation and purification much simpler.

### Enzyme Purification and Activity Assay

BcChiA1 and BatLPMO10 were purified from the culture supernatant via affinity chromatography on Ni-NTA columns and the purity was confirmed by SDS-PAGE (Figure 2B). The molecular weights of the two purified enzymes were consistent with the fermentation results. The specific activity of BcChiA1 with CC-RBB as substrate was 56.16 ± 0.92 U/mg (Figure 2C), which was much higher than the results obtained with other chitinases in previous studies. For example, PbChi70 from *Paenibacillus barengoltzii* displayed a specific activity of 30.3 U/mg (Yang et al., 2016), Chit42 from *Trichoderma harzianum* 5.2 U/mg (Kidibule et al., 2018), and Pa-Chi from *Vibrio parahaemolyticus* 1.5 U/mg (Kadokura et al., 2007).

### Analysis of Changes in Chitin Micro-Structure

**FIGURE 3:**
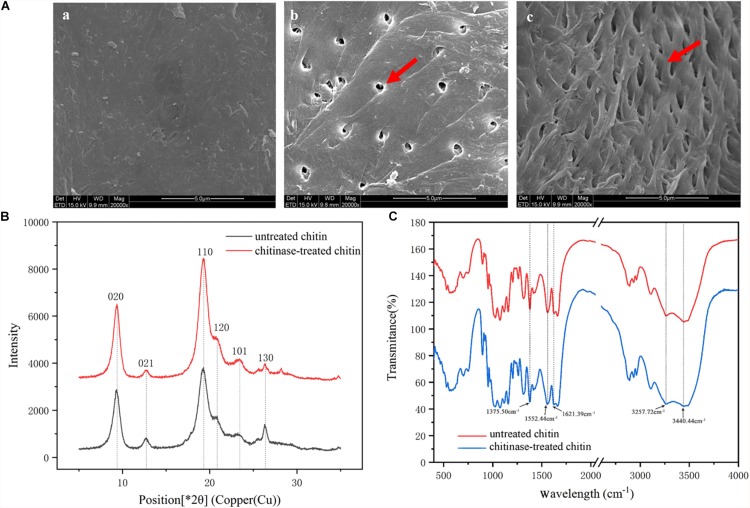
Microstructure of chitin treated with BcChiA1. **(A)** SEM images of **(a)** untreated chitin powder from shrimp shells, **(b,c)** chitin powder treated with BcChiA1. **(B)** XRD curves of untreated chitin powder from shrimp shells (red curve) and chitin powder treated with BcChiA1 (black curve). **(C)** FT-IR spectrometry measurements of untreated chitin powder from shrimp shells (red curve) and chitin powder treated with BcChiA1 (blue curve).

The morphology of chitin powder from shrimp shells before and after chitinase treatment was studied by SEM, as shown in Figure 3A. There was a significant change in the surface topography of chitin powder following chitinase treatment. A rough surface without any interstice could be observed in the untreated chitin powder samples (Figure 3Aa), while the morphology of the samples treated with BcChiA1 showed a porous structure (Figure 3Ab) and high-density fibers (Figure 3Ac). The SEM results indicated that efficient enzymatic degradation of crystalline chitin was achieved via BcChiA1 treatment.

The XRD spectrum of the chitin powder after chitinase treatment was investigated to determine crystal structure and crystallinity (Figure 3B). The position and relative intensity of all the diffraction peaks of the chitinase-treated samples were consistent with the standard XRD pattern of chitin. Moreover, the crystal-reflection peaks of the samples treated with BcChiA1 were lower than those of the untreated chitin powder, which meant the I_*CR*_ of chitin clearly decreased after chitinase treatment. It was calculated that the crystallinity of chitin decreased by 11.84%.

The FT-IR spectra of the chitin powder after chitinase treatment presented in Figure 3C shows characteristic spectral bands of α-chitin at 1660 cm^–1^ and 1625 cm^–1^ (amide I, intra-chain hydrogen bonds with NH groups and inter-chain hydrogen bonds with the primary OH, respectively), 1558 cm^–1^ (amide II), 3266 cm^–1^ (N-H stretching vibrations), and 3443 cm^–1^ (O-H-stretching band) (Xu et al., 2018). The absorption bands of chitin powder were similar to those of the chitinase-treated samples, which meant that the enzymatic degradation did not cause chitin deacetylation.

### Synergistic Effect of BcChiA1 and BatLPMO10 on Chitin Degradation

Recent studies have revealed that LPMOs are active on crystalline polysaccharides and have a synergistic effect with hydrolytic enzymes to stimulate the degradation of crystalline biopolymers (Courtade and Aachmann, 2019). To investigate the influence of BatLPMO10 on the chitin-degradation efficiency of BcChiA1, different concentrations of purified BatLPMO10 were added to preprocess the substrate, and purified BcChiA1 was added to the reaction mixture later (the group without BcChiA1 served as a control). We concluded that BatLPMO10 has practically no ability to degrade chitin wastes independently, as the absorbance at 595 nm barely increased compared with other groups, and was essentially the same as the blank control (with no addition of enzymes). Moreover, the results showed that there was a conspicuous positive correlation between the added amount of BatLPMO10 and the amount of RBB released (absorbance at 595 nm), which confirmed that BatLPMO10 was effective in substrate pretreatment and made the chitin more accessible to degradation by BcChiA1. In the presence of 192 μM BatLPMO10, 4.5 μM BcChiA1 showed the maximum degradation efficiency, which was improved by 50% (shown in the Supplementary Material). The optimum ratio of BcChiA1 and BatLPMO10 for the synergistic effect offers a theoretical basis for the treatment of chitin waste and the production of COS.

### Directed Evolution of BcChiA1

**FIGURE 4:**
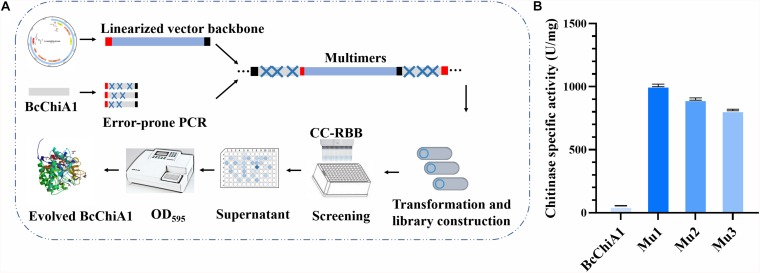
Flow chart of the high-throughput screening based on CC-RBB **(A)** and the specific activity of the three best mutants compared with BcChiA1 **(B)**.

To further improve the efficacy of BcChiA1, directed evolution *in vitro* was applied to the ORF of the BcChiA1 gene without a native signal peptide (Figure 4A). The most important factors in determining the capacity of the mutant library are the quantity of DNA templates, PCR cycle numbers, and the reagent concentration of the Adjustable Error-prone PCR kit. We determined that the optimal conditions encompass 300 ng DNA template, 60 PCR thermal cycles, and 1 μM MnCl_2_. Under these conditions, three rounds of error-prone PCR were conducted, and finally a BcChiA1 mutant library with 12000 clones was constructed. Sixty transformants were randomly selected from the mutagenesis library for DNA sequencing, which revealed an average mutation rate of 0.2%, corresponding to an average of three nucleotide changes within the BcChiA1 gene.

There are three main approaches to chitinase screening in the literature. One relies on transparent zones surrounding colonies on colloidal chitin, the second uses fluorescence based on p-nitrobenzene or 4-methylumbelliferone, and the third is the 3,5-dinitrosalicylic acid (DNS) method. Because all have certain limitations such as low accuracy, low throughput, high cost, poor universality, or cumbersome steps (Fan et al., 2007; Songsiriritthigul et al., 2009; Pan et al., 2019), we established a rapid and sensitive high-throughput screening method based on the substrate CC-RBB, which is non-toxic to microorganisms and can be added directly to growth medium. Using this method, the screening process was greatly shortened. In addition, this method is not restricted by chitinase type and is widely applicable to exo- and endo-chitinases, as well as *N*-acetylglucosaminidases.

After initial high-throughput screening and confirmation via shake-flask fermentation, the three mutants (Mu1, Mu2, and Mu3) were selected, with specific activities of 1004.83 ± 0.87, 906.9 ± 0.55, and 809.36 ± 0.16 U/mg, respectively. These activities corresponded to 16. 89-, 15. 15-, and 13.41-fold increases in activity compared with the wild type (Figure 4B).

### Molecular Dynamic Simulations of the HEI State

Based on the catalytic mechanism of BcChiA1 (Nakamura et al., 2018), Asp163 and Glu165 participate in proton transfer while stabilizing the substrate to complete the catalytic process. Asp163 abstracts a proton from the N atom of the sugar-ring amide, while the carbonyl of the amide attacks the C1 of the sugar ring and forms a pentacyclic structure. At the same time, the O atom attaches to the former sugar ring on the substrate and captures the proton of Glu165 (Figure 1A). Asp163 captures the proton of the substrate, after which the carbonyl attacks the sugar ring. Thus, we retained the newly formed 5-membered ring, the protonated state of Glu165, and the connection of oxygen and C1 of the previous GlcNAc, simulating a transition state before the new bond breaks, as the HEI state (the substrate designated as HEI8, Figure 1B). We constructed the complex between the HEI state of the substrate and BcChiA1 (PDB ID: 1ITX) (Matsumoto et al., 1999) using a restrictive docking method based on the transfer of protons by the substrate and catalytic residues (Figure 1B). Docking studies using the Glide module (Schrödinger, 2018-1) were performed with wild-type bound to HEI8 as the substrate. The structure with d (N6HE8-HD2D163) ≤ 3.5 Å and d (O32HE8-HE2E165) ≤ 3.5 Å (Figure 1B) could be used to represent the start of the hydride transfer process. Therefore, a conformation that satisfies these two constraints can be considered as a sign of the HEI state.

**FIGURE 5:**
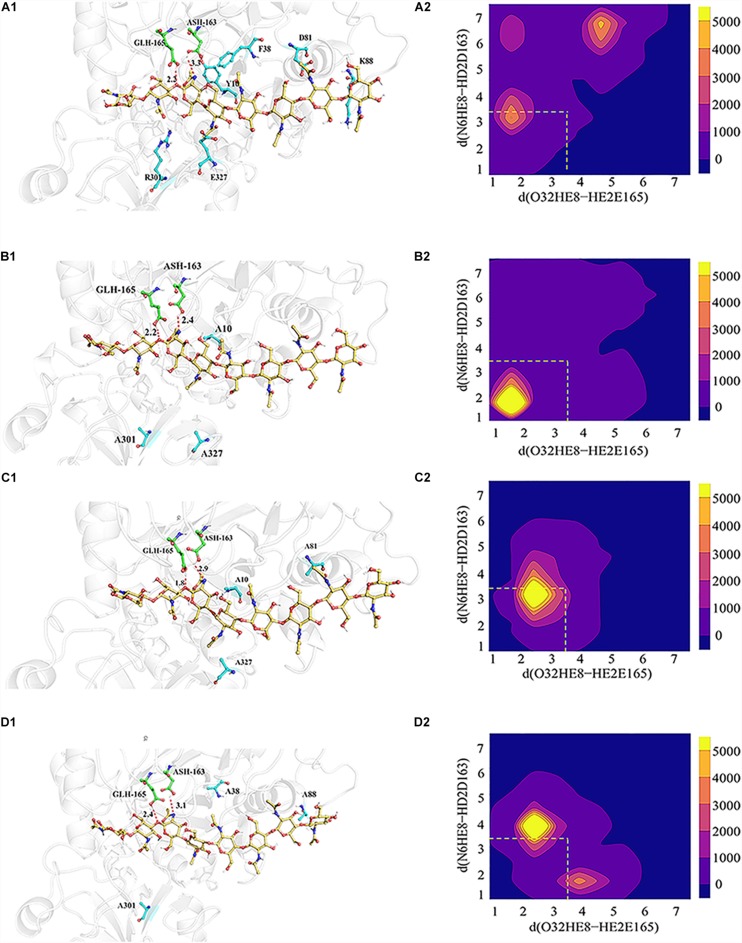
3D model showing the interrelationships between the substrate (yellow), catalytic residues (green) and mutant residues (blue), as well as the statistical distribution of the HEI states throughout the kinetic process. **(A_1_–D_1_)** Signify relative positions of the substrate (yellow), catalytic residues (green) and mutant residues (blue) in WT-HEI8, Mu1, Mu2 and Mu3, respectively. The red dotted line represents the distance of the H atom from two catalytic residues and the O atom and N atom involved in proton transfer, d (O32HE8-HE2E165) and d (N6HE8-HD2D163). **(A_2_–D_2_)** Signify the distributions of these two distances in WT-HEI8, Mu1, Mu2, and Mu3, respectively. Yellow indicates that the occupied frame number in this area is greater than or equal to 5000. For every color change from yellow to blue, the number range of distribution decreases by 1000, whereby pure blue indicates 0. The red dotted box indicates the distribution of the two distances within 3.5 Å.

In order to explore the possible reasons for the enhanced catalytic activity of mutants Mu1, Mu2, and Mu3, MD simulations were performed to evaluate the propensity of the bound substrate to enter the HEI state before and after mutation using ROSETTA3 based on the WT-HEI8 complex. The orientation of HEI8 was observed in the stable HEI states of WT-HEI8 and all the mutants (Figure 5). However, it was difficult to sustain a HEI state of WT-HEI8 (Figure 5A2). Remarkably, the proportions of “catalytic” conformations with both d (N6HE8-HD2D163) ≤ 3.5 Å and d (O32HE8-HE2E165) ≤ 3.5 Å were 79.32, 52.14, and 23.22% in Mu1, Mu2, and Mu3, respectively (Figure 5). Moreover, the frequency of the best mutant, Mu1, was more than ten times that observed in WT-HEI8 (7.28%). These results illustrate that the three mutants with significantly improved activity have active-site structures that are more favorable for the formation of HEI states, which was consistent with the trend of enhanced catalytic activity.

### HPLC Analysis of the Products of Chitin Hydrolysis by BcChiA1

**FIGURE 6:**
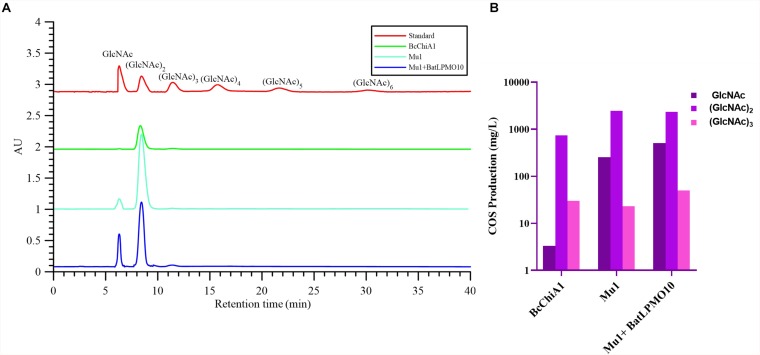
HPLC analysis **(A)** and the yields of GlcNAc, (GlcNAc)_2_, (GlcNAc)_3_ **(B)** of the products of chitin hydrolysis by BcChiA1 and its best mutant Mu1, alone and in combination with BatLPMO10.

HPLC was used to detect the degradation products of chitin using COS standards as references, including GlcNAc, (GlcNAc)_2_, (GlcNAc)_3_, (GlcNAc)_4_, (GlcNAc)_5_, and (GlcNAc)_6_, with respective retention times of 6.303, 8.442, 11.433, 15.742, 21.655, and 30.094 min (Figure 6A). After treatment with BcChiA1, the degradation products GlcNAc, (GlcNAc)_2_, and (GlcNAc)_3_ were detected, while the higher degree of polymerization forms of GlcNAc were not detectable. The HPLC results revealed that (GlcNAc)_2_ was the major product, with a content of 741.90 ± 2.78 mg/L, followed by (GlcNAc)_3_ and GlcNAc (Figure 6B). According to the results, BcChiA1 displayed a stochastic catalytic mode of action on chitin, with a high production of (GlcNAc)_2_. The accumulation of (GlcNAc)_2_ means that BcChiA1 can hydrolyze the second glycoside bond. In this regard, this property of BcChiA1 is similar to those of previously investigated GH family 18 endochitinases from bacteria, such as those from *Paenibacillus barengoltzii* (Yang et al., 2016) and *Streptomyces albolongus* (Gao et al., 2018), as well as a fungal enzyme from *Myrothecium verrucaria* (Vidhate et al., 2019). The best mutant obtained by the directed evolution of BcChiA1, Mu1, was also investigated. The contents of GlcNAc, (GlcNAc)_2_, and (GlcNAc)_3_ after Mu1 treatment were 253.91 ± 0.64 mg/L, 2444.21 ± 4.12 mg/L, and 23.05 ± 0.65 mg/L, respectively (Figure 6B). These values were 76. 39-, 3. 29-, and 0.77-fold higher than the corresponding values of the wild type. In pursuit of a higher yield of COS, the optimum combination of Mu1 and BatLPMO10 was chosen to degrade chitin synergistically. As the results of HPLC analysis show (Figure 6B), the contents of GlcNAc, (GlcNAc)_2_, and (GlcNAc)_3_ were 152. 96-, 3. 14-, and 1.66-fold higher than those obtained after BcChiA1 treatment alone. The HPLC results therefore confirmed a significant increase in chitinase productivity after optimization. The COS yield of Mu1 and BatLPMO10 in this study was 2.89 g/L, which was nearly 3.25-fold higher than the reported yield obtained using the chitinase from *Bacillus* sp. DAU101 (0.89 g/L) (Pan et al., 2019). In addition, the yield of (GlcNAc)_2_ obtained using Mu1 was approximately 2.44 g/L, which was a 20 percent improvement compared with an optimized cocktail of chitinolytic enzymes from *Serratia marcescens* (SmChiA-M, SmChiB, and SmChiC) (Chu et al., 2019). Thus, the combination of evolved BcChiA1 with BatLPMO10 has great practical value.

## Conclusion

BcChiA1 was cloned and its secretory expression was achieved in *B. subtilis* with high efficiency. Furthermore, three improved variants of BcChiA1 were obtained by directed evolution based on an innovative CC-RBB high-throughput screening technique. In addition to the successful optimization of expression level and catalytic activity of BcChiA1 itself, we also found that there is synergy between BcChiA1 and BatLPMO10 during chitin degradation. After directed evolution and optimized combination conditions, the specific activity of the mutant Mu1 reached 1004.83 ± 0.87 U/mg and COS production by a combination of Mu1 and BatLPMO10 was improved up to 2.89 g/L. Most importantly, this study created a novel method for high-throughput screening of chitinase efficiency and provides a new strategy of chitinaceous waste biodegradation in a green and sustainable way.

## Data Availability Statement

All datasets generated for this study are included in the article/Supplementary Material.

## Author Contributions

SW participated in investigation, data curation, writing and original draft preparation. GF, HF, and XW contributed to project administration. JlL and JpL were responsible for methodology, software and formal analysis. DH and DZ took charge of conceptualization and funding acquisition. All authors provided critical advice for the final manuscript.

## Conflict of Interest

The authors declare that the research was conducted in the absence of any commercial or financial relationships that could be construed as a potential conflict of interest.
