# Supplementary material for: High-Efficiency Secretion and Directed Evolution of Chitinase BcChiA1 in Bacillus subtilis for the Conversion of Chitinaceous Wastes Into Chitooligosaccharides
Source: Front Bioeng Biotechnol. 2020 May 7;8:432. doi: 10.3389/fbioe.2020.00432 (PMC7221128; doi:10.3389/fbioe.2020.00432)
Supplement: Supplementary file 1 [file Table_1.DOCX]

Supplementary materials

**Supplementary Table 1. List of all the bacterial strains and plasmids used in this study**

| Strain or plasmid | | | Relevant characteristics | | Sources | |
| --- | --- | --- | --- | --- | --- | --- |
| Strains | *E. coli* DH5α | F−∆*lac*U169(Ø80d *lac*Z∆M15) *sup*E44 *hsd*R17 *rec*A1 *gyr*A96 *end*A1 *thi*-1 *rel*A1 | | Invitrogen | |  |
|  | *B. subtilis*168 | *trpC2* | | Lab stock | |  |
|  | *B. subtilis* 1A751 | *egl*S∆102 *bgl*T/*bgl*S∆EV *apr*E *npr*E *his* | | BGSC | |  |
| Plasmids | pMATE | pMA5 derivative, P*_mal_A* | | Lab stock | |  |
|  | pMATESP0 | pMATE derivative, no SP-BcchiA | | This work | |  |
|  | pMATE SP1 | pMATE derivative, SP_phoD_-BcchiA | | This work | |  |
|  | pMATE SP2 | pMATE derivative, SP_lipA_-BcchiA | | This work | |  |
|  | pMATE SP3 | pMATE derivative, SP_ywbN_-BcchiA | | This work | |  |
|  | pMATE SP4 | pMATE derivative, SP_nprE_-BcchiA | | This work | |  |
|  | pMATE SP5 | pMATE derivative, SP_sacB_-BcchiA | | This work | |  |
|  | pMATE SP6 | pMATE derivative，SP_yvcE_-BcchiA | | This work | |  |
|  | pMATESP7 | pMATE derivative, no SP-BatLPMO10 | | This work | |  |
|  | pMATE SP8 | pMATE derivative, SP_phoD_-BatLPMO10 | | This work | |  |
|  | pMATE SP9 | pMATE derivative, SP_lipA_-BatLPMO10 | | This work | |  |
|  | pMATE SP10 | pMATE derivative, SP_ywbN_-BatLPMO10 | | This work | |  |
|  | pMATE SP11 | pMATE derivative, SP_nprE_-BatLPMO10 | | This work | |  |
|  | pMATE SP12 | pMATE derivative, SP_sacB_-BatLPMO10 | | This work | |  |
|  | pMATE SP13 | pMATE derivative，SP_yvcE_-BatLPMO10 | | This work | |  |

BGSC *Bacillus* Genetic Stock Center, USA

**Supplementary Table 2. List of all the primers sequences used in this study**

| Primer | Sequence (5’→3’) |
| --- | --- |
| pMATESP0-vectorF | tggcagcttcaacatcaccatcatcatcattaaagaagtctcgttccgacagttgg |
| pMATESP0-vectorR | ataatagcccacgatcttatatgaatctgccataattattccccctagctaattttcg |
| pMATESP0-FragmentF | gcagattcatataagatcgtgggct |
| pMATESP0-FragmentR | atgatgatgatggtgatgttgaagc |
| pMATESP1-6-vectorF | GCAGATTCATATAAGATCGTGGGC |
| pMATESP1-6-vectorR | AATTATTCCCCCTAGCTAATTTTCG |
| pMATESP1/8-FragmentF | attagctagggggaataattATGGCATACGACAGTCGTTTTG |
| pMATESP1-FragmentR | acgatcttatatgaatctgcAGCATTTACTTCAAAGGCCCC |
| pMATESP2/9-FragmentF | attagctagggggaataattATGAAATTTGTAAAAAGAAGGATCATTG |
| pMATESP2-FragmentR | acgatcttatatgaatctgcAGCGGCTTTTGCTGACGG |
| pMATESP3/10-FragmentF | attagctagggggaataattATGAGCGATGAACAGAAAAAGCC |
| pMATESP3-FragmentR | acgatcttatatgaatctgcCGCAACGGCTGCCCCCGC |
| pMATESP4/11-FragmentF | attagctagggggaataattGTGGGTTTAGGTAAGAAATTGTCTGTT |
| pMATESP4-FragmentR | acgatcttatatgaatctgcAGCCTGAACACCTGGCAGG |
| pMATESP5/12-FragmentF | attagctagggggaataattATGAACATCAAAAAGTTTGCAAAAC |
| pMATESP5-FragmentR | acgatcttatatgaatctgcCGCAAACGCTTGAGTTGCG |
| pMATESP6/13-FragmentF | attagctagggggaataattGTGAGAAAGAGTTTAATTACACTTGGTTT |
| pMATESP6-FragmentR | ACGATCTTATATGAATCTGCCGCCGATGCAGTTTTACTTGT |
| pMATESP7-vectorF | CACCACCATCACCACtaaagaagtctcgttccgacagttg |
| pMATESP7-vectorR | TCTGATGAATCCTTTcataattattccccctagctaattttcg |
| pMATESP7-FragmentF | agctagggggaataattatgAAAGGATTCATCAGAGGCGTGGTG |
| pMATESP7-FragmentR | gtcggaacgagacttctttaGTGGTGATGGTGGTGGTGTTTAATG |
| pMATESP8-13-vectorF | AAAGGATTCATCAGAGGCGTGG |
| pMATESP8-13-vectorR | AATTATTCCCCCTAGCTAATTTTCG |
| pMATESP8-FragmentR | acgcctctgatgaatcctttAGCATTTACTTCAAAGGCCCC |
| pMATESP9-FragmentR | ACGCCTCTGATGAATCCTTTAGCGGCTTTTGCTGACGG |
| pMATESP10-FragmentR | acgcctctgatgaatcctttCGCAACGGCTGCCCCCGC |
| pMATESP11-FragmentR | acgcctctgatgaatcctttAGCCTGAACACCTGGCAGG |
| pMATESP12-FragmentR | acgcctctgatgaatcctttCGCAAACGCTTGAGTTGCG |
| pMATESP13-FragmentR | acgcctctgatgaatcctttCGCCGATGCAGTTTTACTTGT |

**Supplementary Table 3. List of all the signal sequences used in this study**

| No. | | SP | | Sequences | Length  (aa) | |
| --- | --- | --- | --- | --- | --- | --- |
| 1 | phoD | | MAYDSRFDEWVQKLKEESFQNNTFDRRKFIQGAGKIAGLSLGLTIAQSVGAFEVNA | | 56 |  |
| 2 | lipA | | MKFVKRRIIALVTILMLSVTSLFALQPSAKAA | | 32 |  |
| 3 | ywbN | | MSDEQKKPEQIHRRDILKWGAMAGAAVA | | 28 |  |
| 4 | nprE | | MGLGKKLSVAVAASFMSLSISLPGVQA | | 27 |  |
| 5 | sacB | | MNIKKFAKQATVLTFTTALLAGGATQAFA | | 29 |  |
| 6 | yvcE | | MRKSLITLGLASVIGTSSFLIPFTSKTASA | | 30 |  |

Supplementary Figure 1. The effect of BatLPMO10 addition at different concentrations on the ability of BcChiA1 to degrade chitin (absorbance at 595 nm) .(1: BatLPMO10 (0 μM) and BcChiA1(0 μM); 2: BatLPMO10 (6μM) and BcChiA1(0 μM); 3: BatLPMO10 (0 μM) +BcChiA1 (4.5 μM); 4: BatLPMO10 (6 μM) + BcChiA1 (4.5 μM); 5: BatLPMO10 (12 μM) + BcChiA1 (4.5 μM); 6: BatLPMO10 (24 μM) + BcChiA1 (4.5 μM); 7: BatLPMO10 (48 μM) +BcChiA1 (4.5 μM); 8: BatLPMO10 (96μM) +BcChiA1 (4.5 μM); 9: BatLPMO10 (192 μM) +BcChiA1 (4.5 μM)).

**Simulation Methodology**

All-atom molecular dynamics simulations were performed using the AMBER16 molecular dynamics package (Case et al. 2016). The bonded and non-bonded description of the interactions between the various atoms was conducted using the AMBER16 force fields which include the ff14SB.redq force field parameters. The ANTECHAMBER module and GAFF2 with AM1-BCC charges (Jakalian et al., 2002) was used to obtain force field parameters for ligands. Initially, we performed a series of energy minimization steps to eliminate any bad contacts in the initially built structures. During the minimization, protein (@CA,O,N,C) were restrained with a harmonic force constant of 20 kcal/mol. The minimization involves 5000 steps of steepest descent followed by 5000 steps using the conjugate gradient method. After the energy minimization, the system was slowly heated up to 300 K in 100 ps MD using 1 fs integration time steps, while restraining the solute with a 20 kcal/mol harmonic force constant. After this, we performed 50 ps NPT equilibration of the structures with no harmonic restraints. Subsequently, 10 ns constrained MD simulations were executed, so that the ligand was in a reasonable position to react. Finally, 100 ns NPT production simulations were performed at 300 K and 1 atm pressure with a 2-fs integration time step. We implemented periodic boundary conditions across the system using a TIP3P water box (Jorgensen et al., 1983). We used the Particle Mesh Ewald (PME) technique integrated into the AMBER package to account for the long-range part of the electrostatic interactions (Pearlman et al., 1995). During the dynamic simulations, all the bonds involving hydrogen were restrained using the SHAKE algorithm (Andersen, 1983). A Langevin thermostat with collision frequency of 1 ps-1 was used to maintain the constant temperature, while the pressure was controlled using an anisotropic Monte-Carlo barostat (Chow and Ferguson, 1995). The accelerated GPU version of PMEMD (Le Grand et al., 2013) was performed on NVIDIA GeForce 10 Series cards. We employed CPPTRAJ (Roe and Cheatham, 2013) functionality of AmberTools (Case et al. 2016) to perform various analyses on the equilibrium MD simulation trajectories.

**Enzyme design of the mutants in ROSETTA3**

A cstfile consists of blocks, and for each interaction between two residues (in this study, the hydrogen bond interaction between E165 and HEI8), there needs to be one block. The information in each block defines constraints between three atoms (O32, C41, C42) on E165 and three atoms (OE2, CD, CG) on HEI8. Then, six parameters can be specified, representing the ligand’s six rigid body degrees of freedom. These parameters were given as one distance, two angles, and three dihedrals. In addition, a resfile including residues that need to be mutated was applied in Rosetta enzymedesign (Leaver-Fay et al., 2011; Richter et al., 2011). Based on each cstfile and resfile, an optimized design was implemented using the Rosetta enzymedesign approach and the following command line parameters were used: -enzdes -detect_design_interface -cut1 0.0 -cut2 0.0 -cut3 10.0 -cut4 12.0 -cst_opt -chi_min -bb_min -cst_min -cst_design -design_min_cycles 2-lig_packer_weight1.8-packing:use_input_sc-packing:soft_rep_design-packing: linmen_li 10-nstruct 20. All of them were written in a “flagfile”. The mutants with the best total_score were applied in the MD simulations.

**Reference**

Andersen, H. C. (1983). Rattle: A “velocity” version of the shake algorithm for molecular dynamics calculations. *Journal of Computational Physics,* 52, 24-34. doi: 10.1016/0021-9991(83)90014-1

Case, D., Betz, R., Cerutti, D., Cheatham, T. I., Darden, T., Duke, R., et al. (2016). AMBER 16. *University of California, San Francisco*.

Chow, K.-H., and Ferguson, D. M. (1995). Isothermal-isobaric molecular dynamics simulations with Monte Carlo volume sampling. *Computer Physics Communications,* 91, 283-289. doi: 10.1016/0010-4655(95)00059-O

Jakalian, A., Jack, D. B., and Bayly, C. I. (2002). Fast, efficient generation of high-quality atomic charges. AM1-BCC model: II. Parameterization and validation. *J Comput Chem,* 23, 1623-1641. doi:10.1002/jcc.10128

Jorgensen, W. L., Chandrasekhar, J., Madura, J. D., Impey, R. W., and Klein, M. L. (1983). Comparison of simple potential functions for simulating liquid water. *The Journal of Chemical Physics,* 79, 926-935. doi:10.1063/1.445869

Le Grand, S., Götz, A. W., and Walker, R. C. (2013). SPFP: Speed without compromise—A mixed precision model for GPU accelerated molecular dynamics simulations. *Computer Physics Communications,* 184, 374-380. doi: 10.1016/j.cpc.2012.09.022

Leaver-Fay, A., Tyka, M., Lewis, S. M., Lange, O. F., Thompson, J., Jacak, R., et al. (2011). ROSETTA3: an object-oriented software suite for the simulation and design of macromolecules. *Methods Enzymol,* 487, 545-574. doi:10.1016/b978-0-12-381270-4.00019-6

Pearlman, D. A., Case, D. A., Caldwell, J. W., Ross, W. S., Cheatham, T. E., DeBolt, S., et al. (1995). AMBER, a package of computer programs for applying molecular mechanics, normal mode analysis, molecular dynamics and free energy calculations to simulate the structural and energetic properties of molecules. *Computer Physics Communications,* 91, 1-41. doi: 10.1016/0010-4655(95)00041-D

Richter, F., Leaver-Fay, A., Khare, S. D., Bjelic, S., and Baker, D. (2011). De novo enzyme design using Rosetta3. *PLoS One,* 6: e19230. doi:10.1371/journal.pone.0019230

Roe, D., & Cheatham, T. (2013). PTRAJ and CPPTRAJ: Software for processing and analysis of molecular dynamics trajectory data. *Journal of Chemical Theory and Computation,* 9, 3084-3095. doi:10.1021/ct400341p
